# Two-Year Outcomes of Sapropterin Treatment in Children with Phenylketonuria: A Longitudinal Observational Study of Metabolic, Dietary, and Psychosocial Effects

**DOI:** 10.3390/nu18030446

**Published:** 2026-01-29

**Authors:** Ozlem Yilmaz Nas, Catherine Ashmore, Maria Ines Gama, Anne Daly, Sharon Evans, Alex Pinto, Yahya Ozdogan, Anita MacDonald

**Affiliations:** 1 Birmingham Women’s and Children’s Hospital, Birmingham B4 6NH, UK; catherine.ashmore@nhs.net (C.A.); maria.gama1@nhs.net (M.I.G.); a.daly3@nhs.net (A.D.); evanss21@me.com (S.E.); alex.pinto@nhs.net (A.P.); anita.macdonald@nhs.net (A.M.); 2 Department of Nutrition and Dietetics, Ankara Yildirim Beyazit University, Ankara 06760, Turkey; yozdogan@aybu.edu.tr

**Keywords:** sapropterin, diet, caregiver burden, phenylketonuria

## Abstract

**Background:** Evidence on the long-term impact of sapropterin in phenylketonuria (PKU) is limited. Understanding its effects on dietary restrictions, growth in children, and caregiver burden is essential to optimize PKU management. **Methods:** This prospective, two-year longitudinal study with a comparison group followed 33 children with PKU after sapropterin responsiveness assessment (21 responsive, 12 non-responsive). Outcomes included metabolic control, prescribed protein intake, dietary patterns, growth, psychological measures, and caregiver burden. **Results:** Sapropterin-responsive children increased natural protein intake from 10 g to 28 g/day at 2 years (*p* < 0.001), with reduced protein substitute intake (60 g [56–63] to 45 g [40–60], *p* < 0.05); no changes occurred in non-responsive children (*p* > 0.05). Animal-based foods (cheese, eggs, meat, fish) were introduced in 52% (11/21) of responsive children once tolerance exceeded approximately 25 g/day. The caregivers of responsive children reported reduced financial, familial-social, and personal burden (all *p* ≤ 0.05), alongside decreased food neophobia (*p* = 0.005) and caregiver depression (*p* = 0.013). In sapropterin-responsive children, weight and BMI z-scores remained stable, while height z-score increased over 24 months (*p* = 0.03); non-responsive children had higher weight and BMI z-scores than responsive children at 24 months (*p* = 0.037 and *p* = 0.026). Blood phenylalanine concentrations remained within recommended target ranges overall, with lower median values in responsive children at several time points. **Conclusions:** Sapropterin enabled more flexible, sustainable dietary management in responsive children with PKU, supporting metabolic control, growth, and improved family well-being and social participation. Equitable access to therapies and long-term dietetic support remain essential to optimize outcomes.

## 1. Introduction

Phenylketonuria (PKU) is an autosomal recessive metabolic disorder caused by deficiency of phenylalanine hydroxylase (PAH), leading to accumulation of phenylalanine (Phe) in the blood and brain. First described in the 1930s, PKU became one of the earliest inherited metabolic disorders in which early dietary intervention prevented severe neurocognitive impairment [1,2]. With the introduction of newborn screening, most infants are diagnosed in early life, aiming to begin treatment within the first ten days in order to support normal neurodevelopment [3,4].

Lifelong dietary management remains the foundation of treatment for the majority of people with PKU. They are required to follow a strict low-protein diet that excludes most protein-containing foods and is supplemented with low-Phe protein substitutes, typically fortified with essential micronutrients. Special low-protein foods are incorporated to meet energy requirements and broaden dietary variety [5]. Although this regimen effectively lowers blood Phe, it is complex, socially restrictive, and difficult to sustain. In childhood, challenges are particularly evident in toddlerhood and adolescence, the latter when autonomy and social eating become increasingly important [6,7]. Furthermore, even with good adherence, dietary treatment alone does not fully address neurocognitive difficulties, psychosocial well-being, or quality of life, and adherence generally declines with age [8,9,10,11,12,13].

Over the past two decades, the treatment landscape for PKU has broadened with the introduction of adjunct pharmaceutical therapies. Sapropterin dihydrochloride, sepiapterin, and pegvaliase have each demonstrated the capacity to improve metabolic control, increase natural protein intake, and enhance quality of life [14,15]. Sapropterin, a synthetic analogue of the PAH cofactor tetrahydrobiopterin (BH4), received approval from the U.S. Food and Drug Administration (FDA) in 2007 and the European Medicines Agency (EMA) in 2008 for BH4 responsive PKU, initially restricted to children aged four years and older and subsequently extended to all age groups [16,17]. In the United Kingdom (UK), sapropterin was commissioned by the English National Health Service (NHS) for routine use across all ages in 2021. By augmenting residual PAH activity, sapropterin achieves efficacy in approximately 25–50% of patients, particularly those with milder PAH variants [17,18,19,20]. The British Inherited Metabolic Disease Group (BIMDG) defines long-term responsiveness as either a doubling of natural protein tolerance or improved blood Phe control for those with previous poor control with more than 75% of blood Phe concentrations within therapeutic target range [21].

Sapropterin responsiveness has been consistently associated with improved metabolic control and increased dietary flexibility. Multiple clinical trials and observational studies have demonstrated that responsive individuals achieve higher natural protein tolerance compared with non-responders [16,22,23,24,25,26]. A systematic review of long-term cohorts reported two- to four-fold increases in natural protein tolerance, highlighting the sustained benefit of sapropterin therapy [27]. Furthermore, in the SPARK Phase IIIb extension study, which enrolled children younger than four years, dietary Phe tolerance increased by a mean of 5.5 mg/kg/day from baseline. Importantly, this improvement was accompanied by normal growth trajectories and neuromotor development, supporting both the efficacy and safety of sapropterin in early childhood [16].

However, most Phase I to III studies have been conducted under controlled conditions and of limited duration, providing little insight into real-world dietary behaviour, long-term nutritional adequacy, or broader psychosocial outcomes. Evidence regarding health-related quality of life remains inconsistent, likely reflecting the limited sensitivity of generic questionnaires; however, qualitative reports describe meaningful daily improvements in family routines and dietary flexibility [6].

Dietary adaptations following sapropterin therapy are not well characterized, though available data suggest increased consumption of familiar staple foods such as potatoes, rice, and bread, accompanied by reduced reliance on special low-protein products [28,29]. Concerns have been raised that improved natural protein tolerance may prompt premature discontinuation of protein substitutes, potentially leading to nutritional inadequacy if natural protein sources fail to provide sufficient essential nutrients. In a systematic review, 51% of responsive patients discontinued protein substitutes, with reported reductions in intake of key micronutrients [27]. Growth outcomes appear generally reassuring, with weight and height z-scores remaining within the normal range and some improvement in linear growth when natural protein intake increased substantially [27]. Conversely, Rodrigues et al. [30] reported higher body mass index (BMI) values in sapropterin-treated patients compared with those managed by diet alone.

Understanding the long-term effects of sapropterin on dietary patterns, growth and caregiver burden and well-being is therefore essential for optimizing clinical practice in PKU. This investigation forms part of a two-year prospective, longitudinal, observational study in children with PKU. At the six-month follow-up, Gama et al. [29] reported significant improvements in dietary patterns, food behaviours, and caregiver burden among sapropterin-responsive children with PKU. Natural protein intake increased significantly (*p* < 0.001), while reliance on protein substitutes (*p* = 0.002) and special low protein foods decreased. Caregivers described reduced time spent on PKU-related tasks, lower levels of anxiety and depression, and greater flexibility in social and family eating. The present 24-month prospective follow-up study extends these observations by systematically evaluating long-term clinical, dietary, metabolic, and psychosocial outcomes in both sapropterin-responsive and non-responsive children with PKU.

## 2. Materials and Methods

### 2.1. Study Design and Participants

This single-centre, longitudinal, prospective observational study with a comparison group was conducted at Birmingham Children’s Hospital (BCH), UK, between January 2022 and December 2024. Participants were children aged 3–17 years with a confirmed diagnosis of PKU, or dihydropteridine reductase (DHPR) deficiency. Inclusion criteria comprised children identified through newborn screening with either PAH or DHPR mutations and managed with a Phe-restricted diet. Exclusion criteria included late diagnosis of PKU, the presence of comorbid conditions unrelated to PKU (e.g., diabetes), and teenage pregnancy.

As sapropterin responsiveness reflects underlying PAH genotype and phenotype severity, the responsive and non-responsive groups were not biologically equivalent at baseline. Children with DHPR deficiency were included because they follow the same Phe restricted dietary management and sapropterin testing pathway as PAH related PKU.

All participants were managed within the British Inherited Metabolic Disease Group (BIMDG) pathway [21] as part of routine clinical care. Sapropterin responsiveness testing involved establishing a pre-sapropterin baseline using six early morning fasting dried blood spots collected over ≤2 weeks. Sapropterin was administered at 20 mg/kg/day with fasting dried blood spot sampling conducted for a further 6 days in a 28 day trial. A mean ≥30% reduction in blood Phe from the pre-sapropterin baseline defined biochemical responsiveness. Participants were subsequently allocated to one of two groups based on sapropterin responsiveness. Individuals were classified as sapropterin non-responsive if they had either two null PAH variants or demonstrated less than a 30% reduction in blood Phe concentrations during the sapropterin trial. They continued their standard Phe-restricted diet and usual protein substitute dosage.

Those achieving a reduction of 30% or more in blood Phe with a subsequent increase in Phe tolerance of 100% were categorized as sapropterin responsive.

### 2.2. Data Collection

**Figure 1:**
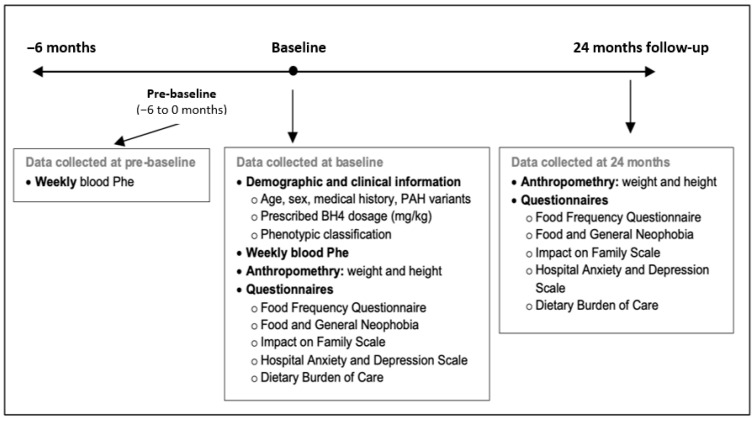
Study timeline and data collection.

Data were collected at two primary time points: baseline (defined as sapropterin initiation for BH4-responsive participants or the equivalent assessment for non-responsive participants) and at 24 months. Retrospective blood Phe concentrations from the six months preceding baseline (pre-baseline) were also collected and analyzed to evaluate pre-treatment metabolic control. The overall study timeline and data collection points are summarized in Figure 1.

Demographic and clinical information, including age, sex, relevant medical history, PAH genetic variants, and prescribed sapropterin dosage (mg/kg), was extracted from hospital medical records. Phenotypic classification was determined based on pre-sapropterin Phe tolerance. Anthropometric measurements, including weight and height were performed by trained healthcare professionals and recorded in hospital databases.

At baseline and at 24 months, caregivers completed all questionnaires, and adolescents aged 12 years or older completed the Hospital Anxiety and Depression Scale (HADS). Questionnaires were administered either during clinic visits or at home by a specialist metabolic dietitian experienced in PKU management.

### 2.3. Biochemical Assessment

Fasting morning dried blood spot (DBS) samples for Phe quantification were obtained at home by caregivers on a weekly schedule, using standardized filter cards (Perkin Elmer 226; UK Standard Newborn Screening). All caregivers received structured training in DBS collection and handling procedures from specialist metabolic nurses prior to study participation. Completed DBS cards were forwarded to the hospital laboratory via first-class postal service and subsequently analyzed by tandem mass spectrometry (MS/MS).

### 2.4. Anthropometric Measurements

Weight (kg) and height (cm) were measured at each research visit using calibrated SECA^®^ medical measuring systems (SECA 875 scales and SECA 213 stadiometer, Hamburg, Germany) to the nearest 0.1 kg and 0.1 cm, respectively. Measurements were obtained by trained metabolic dietitians with participants wearing light clothing and no shoes. Age- and sex-specific z-scores for weight, height, and BMI (kg/m^2^) were calculated using World Health Organization (WHO) growth reference data [31].

### 2.5. Questionnaires

Food Frequency Questionnaire (FFQ): dietary intake was assessed using a validated 89-item FFQ specifically developed for PKU [32]. The questionnaire evaluated the frequency and portion size of regular and low-protein foods. Caregivers were provided with a photographic food portion guide to support accurate estimation. Reported portion sizes were converted into weekly frequencies for analysis.

Food and General Neophobia Questionnaire: this tool was adapted from the validated adult neophobia scale developed by Pliner and Hobden [33]. It comprised nine items assessing food neophobia and five items assessing general neophobia. Caregivers rated responses on a seven-point scale ranging from 1 (always) to 7 (never). To maintain scoring consistency, five items reflecting willingness to try new foods (e.g., “My child frequently tries new and different foods”) were reverse-scored. Total scores were computed after recoding, with lower scores indicating higher levels of food and general neophobia.

Impact on Family Scale: family burden was evaluated using a validated 24-item questionnaire assessing the impact of chronic disease management across four domains: financial impact, familial-social impact, personal strain (psychological stress), and mastery (coping) [34]. Each item was scored from 1 (higher impact) to 4 (lower impact), with lower subscale scores indicating greater family burden.

Hospital Anxiety and Depression Scale (HADS): psychological well-being was assessed using the validated 14-item HADS tool [35]. Caregivers and adolescents aged 12 years or older completed the questionnaire, which included seven items each for anxiety and depression, scored on a four-point scale (0–3). Scores of 0–7 indicate normal, 8–10 borderline abnormal, and 11–21 abnormal levels of anxiety or depression.

Dietary Burden of Care Questionnaire: caregiver burden related to dietary management was explored using a non-validated, open-ended questionnaire consisting of 16 items. Questions addressed daily PKU management, emotional responses to sapropterin testing, dietary routines, use of low-protein foods, protein substitute administration, food expenditure, eating out, holiday arrangements, school participation, and social activities. Caregivers were invited to describe practical challenges, coping strategies, and changes in family life following sapropterin testing. Adolescents aged 12 years or older were encouraged to contribute their perspectives when appropriate.

### 2.6. Statistical Analysis

All statistical analyses were performed using Numiqo version 2025 [36]. The distribution of continuous variables was assessed using the Shapiro–Wilk test and visual inspection of Q–Q plots. Normally distributed data are presented as mean ± standard deviation (SD), and non-normally distributed data are reported as median and interquartile range (Q1–Q3).

Within-group comparisons between baseline and 24 months were conducted using the paired t-test for normally distributed variables and the Wilcoxon signed-rank test for non-normally distributed variables. Between-group comparisons (sapropterin-responsive versus non-responsive) were performed using independent-samples *t*-tests or Mann–Whitney U tests, as appropriate. Differences in median weekly portions of regular foods across natural protein tolerance categories (<15 g, 15–25 g, 25–40 g, >40 g) were analyzed using the Kruskal–Wallis test, with Bonferroni-adjusted post hoc pairwise comparisons applied when overall differences were significant.

Metabolic control was evaluated across four-time intervals: pre-baseline, 0–6 months, 6–12 months, and 12–24 months. Pre-baseline was defined as the mean of all blood Phe concentrations obtained during the six months preceding baseline. Median blood Phe concentrations were compared between groups at each time point using the Mann–Whitney U test. The therapeutic upper target range for blood Phe was defined according to current European PKU guidelines [37]: 360 µmol/L for participants younger than 12 years and 600 µmol/L for those aged 12 years or older. The proportion of blood Phe results exceeding the upper therapeutic range was analyzed using the Chi-square test. A *p*-value < 0.05 was considered statistically significant.

Responses to open ended questionnaires exploring dietary burden of care were analyzed using an inductive thematic approach. All responses were read multiple times to ensure data familiarization prior to coding. Data were then coded manually, and codes were iteratively reviewed and grouped into overarching themes reflecting shared experiences and contrasting perspectives between sapropterin responsive and non responsive participants. Frequencies (*n*, %) were calculated for recurring themes to provide descriptive comparisons between groups, and representative verbatim quotations were selected to illustrate key findings.

### 2.7. Ethics

The study protocol was approved by the UK Wales Research Ethics Committee (REC reference: 22/WA/0143; IRAS ID: 314071) and received institutional research and development approval from Birmingham Women’s and Children’s NHS Foundation Trust. The study was conducted in accordance with the Declaration of Helsinki, UK legislation, and Good Clinical Practice guidelines. Written informed consent was obtained from caregivers, and assent was sought from children when appropriate.

## 3. Results

### 3.1. Participants

A total of 33 children with PKU were enrolled in the study (*n* = 16 male; 48%). Of these, 21 (64%) were classified as sapropterin-responsive and 12 (36%) as non-responsive. The mean age was 10.1 ± 4.2 years in the sapropterin-responsive group and 10.4 ± 3.8 years in the non-responsive group. Genetic analysis was available for 27 children (82%). From pre-sapropterin Phe tolerance, 24 (73%) were identified as classical PKU, 7 (21%) with mild PKU, and 2 (6%) with DHPR deficiency (Supplementary Table S1). Sapropterin-responsive children received once-daily sapropterin at a median dose of 20 mg/kg (range 10 to 20 mg/kg); both children with DHPR deficiency were treated with sapropterin at 20 mg/kg/day, and received neurotransmitter precursor therapy. Two sapropterin-responsive children had a coexisting diagnosis of autism spectrum disorder. One 16 year old sapropterin-responsive participant was non-adherent with dietary treatment following sapropterin treatment and was excluded from dietary protein intake analyses only.

### 3.2. Metabolic Control

A total of 3816 blood Phe measurements were analyzed during the study period (Table 1).

Among children aged <12 years in both sapropterin-responsive and non-responsive groups (*n* = 19; mean age: 7.2 ± 2.0 years), median blood Phe concentrations at baseline and at 24 months remained within the therapeutic target range (120 to 360 µmol/L) [37]. At both time points, sapropterin-responsive children exhibited lower median Phe concentrations than non-responsive children (baseline: 270 vs. 310 µmol/L; 24 months: 240 vs. 285 µmol/L; all *p* < 0.05). The percentage of blood Phe samples exceeding 360 µmol/L was also lower in the responsive group at baseline and at 24 months (11% and 16%, respectively) compared with the non-responsive group (29% and 37%, respectively), with significant differences observed at both time points (all *p* < 0.001).

Among participants aged ≥12 years in both groups (*n* = 14; mean age: 14.3 ± 1.7 years), median blood Phe concentrations at baseline and at 24 months were higher overall than in younger children but remained within the age-specific therapeutic range (120 to 600 µmol/L). At both time points, sapropterin-responsive adolescents demonstrated lower median Phe concentrations compared with non-responsive adolescents (baseline: 455 vs. 540 µmol/L [*p* < 0.001]; 24 months: 370 vs. 600 µmol/L; [*p* < 0.001]). During adolescence, episodes of elevated blood Phe (>600 µmol/L) were observed in both sapropterin-responsive and non-responsive groups. However, high-Phe concentrations occurred significantly more frequently among non-responsive adolescents during the 12–24 month period (27% vs. 47%, *p* < 0.001).

### 3.3. Prescribed Protein Intake

Table 2 summarizes the prescribed protein intake at baseline and 24 months. One sapropterin-responsive patient who was non-adherent and discontinued dietary treatment was excluded from the protein intake analysis. Among the remaining sapropterin-responsive group, natural protein intake increased significantly from a median of 10 g/day (Q1–Q3: 6–13) at baseline to 28 g/day (Q1–Q3: 20–40) at 24 months (*p* < 0.001), whereas no significant change was observed in the non-responsive group (*p* > 0.05). At 24 months, the natural protein intake in the responsive group ranged from 10 to 65 g/day and was significantly higher compared with the non-responsive group (*p* < 0.001). In contrast, no significant change in natural protein intake was observed in the non-responsive group (*p* > 0.05).

Protein equivalent intake from protein substitutes decreased significantly in the sapropterin-responsive group (*p* < 0.05) and remained unchanged in the non-responsive group (*p* > 0.05). In one sapropterin-responsive child, protein substitute intake was fully discontinued. One adolescent who ceased protein substitute use due to poor adherence was excluded from this analysis. At 24 months, between-group comparison demonstrated higher protein substitute intake in the non-responsive group (*p* < 0.001).

### 3.4. Anthropometric Changes

Table 3 presents anthropometric outcomes at baseline and 24 months. Anthropometric measures remained stable in the sapropterin-non-responsive group over time (within-group *p* > 0.05). In the sapropterin-responsive group, weight and BMI z-scores remained stable over the 24-month follow-up, while height z-score increased significantly from baseline to 24 months (*p* = 0.03). At 24 months, the sapropterin-non-responsive group had significantly higher weight and BMI z-scores compared with the responsive group (*p* = 0.037 and *p* = 0.026, respectively). Height z-scores did not differ significantly between groups (*p* = 0.515).

### 3.5. Food Intake Patterns

Table 4 shows changes in regular and low-protein food consumption from baseline to 24 months. At 24 months, significant dietary shifts were observed in the sapropterin-responsive group. Consumption of several regular foods increased markedly, including cow’s milk (19% to 43%; *p* = 0.009), regular cheese (14% to 48%; *p* = 0.009), meat, fish and eggs (24% to 52%; *p* = 0.004), regular bread (24% to 81%; *p* = 0.013), and regular pasta (38% to 90%; *p* = 0.001). Correspondingly, intake of low-protein milk, cheese, bread, and pasta decreased significantly (all *p* ≤ 0.01). In contrast, no significant within-group changes were observed in the non-responsive group across any food category (*p* > 0.05). Intake of potato chips and processed potatoes, vegetables, and fruit remained stable over time in both groups (*p* > 0.05).

**Figure 2:**
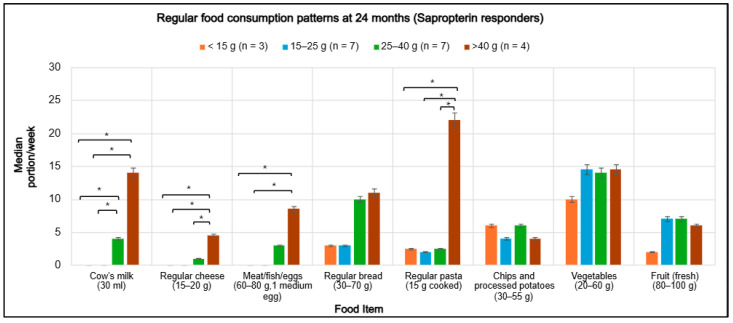
Median weekly portions of regular foods at 24 months among sapropterin-responsive children, stratified by natural protein intake (<15 g, 15–25 g, 25–40 g, >40 g). Error bars represent 95% confidence intervals. Group differences were assessed using the Kruskal–Wallis test, followed by pairwise post hoc comparisons with Dunn’s test and Bonferroni correction. * Significant differences (*p* < 0.05) are indicated by asterisks.

At 24 months, median weekly portions of regular foods varied according to Phe tolerance among sapropterin-responsive children (Figure 2). Higher tolerance was associated with increased consumption of high-protein foods, including cow’s milk, regular cheese, meat, fish, eggs, and pasta. Children with a protein tolerance >25 g/day (25–40 g and >40 g/day groups) consumed substantially more of these foods compared with less tolerant groups. The >40 g/day protein tolerance group demonstrated the highest intake of cow’s milk (14 portions/week; *p* = 0.031), regular cheese (4.5 portions/week; *p* = 0.036), meat, fish, and eggs (8.5 portions/week; *p* = 0.024), and regular pasta (22 portions/week; *p* = 0.029). In contrast, intake of these foods was negligible among responsive children with protein tolerance <15 g/day. Bread consumption increased progressively in line with rising natural protein tolerance, ranging from 3 to 11 portions per week (*p* = 0.39). In contrast, intake of chips and processed potatoes (4–6 portions/week; *p* = 0.82), vegetables (10–14.5 portions/week; *p* = 0.77), and fruit (2–7 portions/week; *p* = 0.96) remained stable across the study period.

### 3.6. Impact on Family

Table 5 summarizes the Impact on Family Scale scores at baseline and 24 months. Lower subscale scores indicate greater family burden, whereas higher scores reflect lower impact.

In the sapropterin-responsive group, financial (*p* = 0.017), familial-social (*p* < 0.001), personal strain (*p* = 0.003), and total scores (*p* < 0.001) increased significantly from baseline to 24 months, consistent with a reduction in family burden. In the non-responsive group, there was a small but significant increase in personal strain scores (*p* = 0.04), while financial, familial-social, mastery (perceived control and coping ability), and total scores remained unchanged (*p* > 0.05).

Between-group comparisons at 24 months showed significantly higher familial-social impact scores in the sapropterin-responsive group compared with the non-responsive group (*p* = 0.01). Differences in financial, personal strain, mastery, and total scores between groups were not statistically significant (*p* > 0.05).

### 3.7. Food Neophobia and Psychological Outcomes

Table 6 summarizes scores from the Food and General Neophobia Questionnaire and the HADS at baseline and 24 months. In the sapropterin-responsive group, food neophobia scores increased significantly between baseline and 24 months (*p* = 0.005), indicating reduced food neophobia over time. General neophobia scores showed no significant change in either group (*p* > 0.05). At 24 months, no significant between-group differences were observed for food neophobia, general neophobia, or any HADS subscales (*p* > 0.05).

HADS anxiety and depression scores remained stable from baseline to 24 months across all participants (*p* > 0.05). Among caregivers in the sapropterin-responsive group, depression scores decreased significantly from 4 (Q1–Q3: 2–7) to 1 (Q1–Q3: 0–3) (*p* = 0.013), whereas anxiety scores showed no change. In contrast, no significant changes were observed in caregivers in the non-responsive group. In adolescents, both anxiety and depression scores remained stable over time in both groups (*p* > 0.05). No significant differences were found between sapropterin-responsive and non-responsive groups for any psychological outcomes (*p* > 0.05).

### 3.8. Caregiver Burden of Care

Table 7 summarizes caregiver experiences 24 months after sapropterin testing, covering emotional impact, dietary management, financial and social aspects, and school-related arrangements. The questionnaire was completed by caregivers, with adolescents contributing their views where appropriate.

#### 3.8.1. Emotional and Practical Impact

Most caregivers of sapropterin-responsive children (20/21, 95%) described sapropterin as life-changing, highlighting improved blood Phe control, greater dietary flexibility, and reduced stress around meal planning. Caregivers frequently mentioned being able to eat out and enjoy spontaneous meals. One caregiver stated, *“Life is just a joy now. We do not worry about food or stress anymore.”* One adolescent added, *“Positive, happy, life changing. I can now go out with friends to a café and have a ‘normal’ packed lunch.”* However, another adolescent who struggled with dietary adherence reflected, *“The drug opened up a new area of food, and then I skipped my protein substitute and ignored my diet.”*

All non-responders (12/12, 100%) reported little or no change in daily life. Many expressed frustration or disappointment with the sapropterin responsiveness test outcome, although most later accepted the result and some expressed hope for future treatment options. One caregiver commented, *“A bit disappointed, even though I knew it had a low chance of working. I still hoped it might work.”*

#### 3.8.2. Dietary Flexibility and Family Life

All caregivers of sapropterin-responsive children (21/21, 100%) reported markedly increased dietary flexibility, describing greater freedom to share family meals and eat more spontaneously. As one caregiver explained, *“Now he can pick food straight off the supermarket shelf. We can have takeaways and go to restaurants without planning ahead.”*

Food preparation time decreased to typical household levels for most caregivers (18/21, 86%), who commonly described relief at *“not needing to plan every meal.”* In contrast, all non-responsive families (12/12, 100%) continued to report rigid dietary restrictions and substantial time demands associated with shopping, weighing, and using low protein foods for meal preparation.

#### 3.8.3. Use of Low-Protein Foods and Protein Substitutes

Use of special low-protein foods decreased in the responsive group, with 14/21 caregivers (67%) reporting rare or no use. One noted, *“We do not need low-protein foods anymore, just occasional top-ups.”* In contrast, all caregivers of non-responsive children (12/12, 100%) continued daily use of low-protein staples such as bread and pasta.

Protein substitutes were generally well tolerated and consumed quickly (under one minute). Two sapropterin-responsive children stopped using protein substitutes, one because it was no longer required and one due to non-adherence. Minor issues were reported in 5/12 (42%) of the non-responsive group, usually when children were unwell.

#### 3.8.4. Financial and Social Aspects

Most caregivers of sapropterin-responsive children (14/21, 67%) reported stable or reduced food costs, mainly because the whole family could share the same meals. As one caregiver described, *“Everyone eats the same food now. It is simpler and more economical.”* A minority (5/21, 24%) reported slightly higher food costs related to increased appetite, consumption of regular foods, or occasional takeaways. In contrast, most caregivers of non-responsive children (9/12, 75%) reported persistently high or rising food expenses.

#### 3.8.5. Social Participation and Eating out

Eating out was common among responsive families (21/21, 100%), typically weekly or monthly. One caregiver commented, *“We can go out whenever we want. No planning is needed.”* Participation in parties or food-related events was also high (18/21, 86%), and sleepovers were permitted for most children (13/21, 62%).

In comparison, only 3/12 (25%) of non-responsive families reported eating out regularly. Social participation was more limited, with 8/12 (67%) attending events occasionally and often requiring advance preparation. One caregiver described, *“Preparation and organisation start months in advance. It is very time consuming and stressful.”*

#### 3.8.6. School and Holidays

School participation, protein substitute use at school, and holidays differed between groups. In the sapropterin responsive group, three participants were not attending school at the time of data collection. Among school attending participants (*n* = 18), packed lunches were reported by 13 (72%), while some children accessed school meals (5/18, 28%). Consistent protein substitute use at school was reported for 76% (13/17). Holidays within the UK or abroad were reported by 10/21 families (48%). One caregiver stated, “*We can go anywhere now. We went abroad twice last year*.”

In the sapropterin non-responsive group, packed lunches were reported for 8/12 participants (67%), and protein substitute use at school was reported consistently in all cases (12/12, 100%). Holidays within the UK or abroad were reported by 7/12 families (58%), with planning and organization required, and travel described as stressful and time intensive.

## 4. Discussion

This two-year prospective, longitudinal observational study evaluated metabolic, dietary, and psychosocial outcomes in sapropterin-responsive versus non-responsive children with PAH-related PKU or DHPR deficiency. It should be noted that the responsive and non-responsive groups were not biologically equivalent at baseline, as sapropterin responsiveness is associated with milder PAH variants; this underlying difference likely explains the higher pre-baseline blood Phe concentrations observed in the non-responsive group and should be considered when interpreting group comparisons. Blood Phe concentrations generally remained within recommended target ranges across both cohorts; however, sapropterin-responsive children demonstrated better metabolic control over 24 months, characterized by lower median Phe levels and fewer peaks above target thresholds. Growth trajectories within groups were largely stable; however, height z-scores increased significantly in sapropterin-responsive children, and non-responsive children had significantly higher weight and BMI z-scores at 24 months. Dietary outcomes diverged markedly between groups. In the sapropterin responsive group, natural protein tolerance increased by approximately 180% over 24 months. In contrast, non-responsive children showed no significant change in natural protein intake and continued to adhere to strict dietary regimens. Family-reported measures reflected these differences. Caregivers of sapropterin-responsive children reported reduced dietary burden and greater flexibility in daily food choices, whereas families of non-responsive children continued to experience high levels of care-related strain. Collectively, these findings highlight the sustained metabolic advantages, dietary liberalization, and psychosocial benefits associated with sapropterin responsiveness.

Dietary patterns repositioned substantially among sapropterin-responsive children as natural protein tolerance increased. Families progressively substituted special low-protein foods with regular foods, reflecting enhanced dietary flexibility and variety. A threshold of approximately 25 g/day of natural protein was associated with the introduction of animal-derived protein containing foods such as meat, fish, and eggs, with intake remaining negligible below this level. Once tolerance exceeded 40 g/day, children demonstrated markedly greater dietary freedom, characterized by more frequent consumption of regular dairy products, meat, fish, eggs, and staple foods, indicative of a more liberal and less restrictive eating pattern. These observations are consistent with current recommendations that dietary liberalization should proceed incrementally: beginning with replacement of modified low-protein staples by regular versions, followed by introduction of easily portioned, high-quality protein sources (e.g., milk, yoghurt, egg, cheese), while reserving meat until tolerance approaches near-unrestricted levels due to its high Phe content [38]. Children appeared to adapt well to the taste and texture of protein-rich foods once permitted, and many expressed a preference to discontinue low-protein alternatives. Importantly, expansion of protein intake did not displace fruit or vegetable consumption, suggesting that dietary diversification was additive rather than substitutive and supporting maintenance of a balanced dietary pattern. These findings are reassuring in light of concerns that reduced reliance on protein substitutes may compromise micronutrient intake or overall diet quality if convenience foods are prioritized [39]. They highlight the importance of clear, staged dietary guidance to safeguard metabolic control during liberalization, reinforcing the role of dietetic-led, planned progression in managing increased natural protein tolerance.

Dietary liberalization in sapropterin-responsive children occurred in parallel with sustained metabolic control. Responsive children consistently maintained lower blood Phe concentrations with fewer values above target thresholds compared with non-responsive peers (*p* ≤ 0.05). Among adolescents, sapropterin treatment was associated with stable metabolic outcomes, whereas non-responsive adolescents demonstrated marked deterioration, with median Phe values approaching the upper therapeutic limit of 600 µmol/L by 12–24 months (*p* < 0.001). During this period, nearly half of Phe measurements in non-responsive adolescents exceeded the therapeutic limit, compared with 27% in responsive peers, emphasizing the clinical importance of responsiveness in mitigating poor metabolic control. These findings corroborate previous evidence that sapropterin therapy can safely and effectively reduce blood Phe concentrations in both short-term [17,40] and long-term studies [26], while enhancing metabolic stability [41]. Collectively, the results highlight the sustained benefits of sapropterin responsiveness for metabolic management and reinforce its role in supporting dietary liberalization without compromising metabolic control.

Growth over two years was reassuring in sapropterin-responsive children, with weight, height, and BMI z-scores remaining within reference ranges and height z-scores significantly improving. This suggests that dietary liberalization, when managed in a structured way, does not compromise nutritional status. These results contrasts with some reports of BMI increases on sapropterin [27,30], though evidence remains inconsistent [42] and generally remains within one standard deviation of norms. Overall, the growth in PKU appeared to be more influenced by baseline nutritional status, diet quality, and lifestyle than by sapropterin itself. These findings reinforce the need for continued nutritional counselling during dietary liberalization and highlight the value of larger, long-term studies incorporating detailed assessments of diet, body composition, and physical activity. In contrast, non-responsive children maintained higher weight and BMI z-scores throughout, reflecting pre-existing trajectories and the greater severity of classical PKU. Higher BMI has previously been reported among individuals with classical PKU, with higher prescribed energy intake to prevent catabolism suggested as a potential explanation [30].

Caregivers of sapropterin-responsive children consistently described treatment as transformative, reflecting a substantial improvement in day-to-day PKU management driven by increased dietary flexibility. Families reported being able to share meals, eat outside the home, and make more spontaneous food choices, thereby reducing the constant planning and preparation traditionally required. These practical benefits translated into significant improvements in family functioning, including reduced financial burden, greater social participation, and lower personal strain. Such findings are consistent with previous reports demonstrating improved quality of life following sapropterin initiation [6,29]. In line with these perceived benefits, caregiver depression scores decreased significantly over 24 months, while caregiver anxiety and both anxiety and depression scores in adolescents remained unchanged. This aligns with earlier literature suggesting that generic instruments may lack sensitivity to detect meaningful, condition-specific changes in PKU, which are more readily captured through qualitative approaches and PKU-specific tools [9,43,44].

Importantly, increased dietary freedom did not equate to unrestricted eating for all sapropterin-responsive children. While most sapropterin responders integrated dietary liberalization successfully, dietary adherence deteriorated in one 16-year-old sapropterin-responsive adolescent who experienced significant psychosocial difficulties. This example underlines that pharmacological responsiveness alone does not guarantee sustained adherence. Age-appropriate, multidisciplinary support is therefore essential to address behavioural and psychosocial challenges as adolescents assume greater responsibility for dietary management.

Families of non-responsive children described little or no change in dietary tolerance over time. Dietary management continued to centre around a strict regimen, with ongoing demands related to food planning, shopping, weighing, and meal preparation. Many caregivers expressed disappointment when the responsiveness outcome was first communicated, followed over time by acceptance, while the practical, financial, and social constraints of dietary treatment persisted. Quantitative measures reflected this stability, with no meaningful improvement across financial, familial-social, or mastery domains and only minimal change in personal strain. Measures of anxiety and depression scores for caregivers and adolescents also remained unchanged. These findings mirror wider evidence that long-term dietary management in PKU carries a sustained psychosocial burden, characterized by reduced flexibility, constrained social participation, and ongoing caregiver responsibility [45,46,47,48]. Whilst sapropterin significantly eased the daily management burden for caregivers of responsive children, the confirmation of non-responsiveness marked a moment of particular vulnerability for families whose children remained dependent on dietary treatment alone. At this transition, caregivers need structured, proactive support to navigate the emotional and practical implications of continued dietary restriction.

This study possesses several important strengths, including its two-year, prospective, longitudinal design with a comparison group and the integrated assessment of metabolic, dietary, and psychosocial outcomes, which together enabled a comprehensive evaluation of sapropterin’s effects. Nonetheless, several limitations should be considered. The single-centre design and relatively small sample size, particularly within the adolescent subgroup, may restrict the generalisability of the findings. In addition, the sample size differed between sapropterin-responsive and non-responsive groups, which may have influenced the robustness of between group comparisons. Dietary intake data were derived from caregiver reported FFQ and are therefore susceptible to recall bias, although families managing PKU typically demonstrate strong proficiency in dietary monitoring. Anthropometric changes should be interpreted with caution, as differences in growth velocity may reflect normal variation in pubertal timing. Because pubertal stage was not documented, we were unable to adjust for prepubertal versus pubertal status. Micronutrient intake data and blood nutritional markers were not reported in the present analysis, limiting the ability to objectively assess dietary sufficiency and nutritional status; these outcomes will be examined in a subsequent publication. The psychological measures employed were standardized rather than condition specific, which may have reduced sensitivity to subtle or PKU specific aspects of lived experience; however, qualitative responses offered valuable contextual insight into everyday challenges and perceived treatment benefits. The dietary burden of care questionnaire had not been formally validated. Finally, developmental changes over the study period and unmeasured confounding variables may also have influenced the observed outcomes.

## 5. Conclusions

This two-year longitudinal study shows that sapropterin responsiveness in children with PKU is associated with improved metabolic control, increased dietary freedom, and reduced burden of care. Increased natural protein tolerance allowed progressive dietary change; however, the extent of dietary liberalization varied between individuals according to their PKU severity. While responsive families experienced greater flexibility in daily routines and food choices, non-responsive families continued to manage the persistant demands of rigid dietary restriction with minimal change over time. These findings highlight the importance of structured dietary planning, ongoing monitoring, and tailored support to maintain metabolic stability and nutritional adequacy, particularly during adolescence when adherence may be challenged by increasing independence. Continued evaluation of long-term dietary quality, psychosocial outcomes, and treatment burden is needed to guide safe implementation of emerging therapies and to support equitable, sustainable care for children and families across the full PKU spectrum.

## Figures and Tables

**Table 1:** Blood Phe concentrations and proportion of samples above the therapeutic target in sapropterin-responsive and non-responsive children over time.

Follow-Up Period	Group	*n*	Blood Phe (µmol/L) Median (Q1–Q3)	*p* ^1^	Samples (*n*)	Above Target n (%) ^3^	*p* ^2^
		Children < 12 years (*n* = 19; mean age: 7.2 ± 2.0 years)					
Pre-baseline (6 months before baseline)	Sapropterin-responsive	13	270 (210–330)	0.018	437	71 (16%)	<0.001
	Sapropterin-non-responsive	6	300 (200–400)		176	52 (30%)	
0–6 months	Sapropterin-responsive	13	240 (200–310)	0.031	413	49 (12%)	<0.001
	Sapropterin-non-responsive	6	295 (178–460)		167	62 (37%)	
6–12 months	Sapropterin-responsive	13	240 (180–310)	<0.001	295	33 (11%)	<0.001
	Sapropterin-non-responsive	6	310 (200–400)		164	48 (29%)	
12–24 months	Sapropterin-responsive	13	250 (190–320)	0.001	485	76 (16%)	<0.001
	Sapropterin-non-responsive	6	285 (188–420)		222	71 (32%)	
		Adolescents ≥ 12 years (*n* = 14 mean age: 14.3 ± 1.7 years)					
Pre-baseline (6 months before baseline)	Sapropterin-responsive	8	420 (280–550)	0.318	182	26 (14%)	0.261
	Sapropterin-non-responsive	6	410 (220–520)		165	17(10%)	
0–6 months	Sapropterin-responsive	8	400 (240–590)	0.136	172	33 (19%)	0.206
	Sapropterin-non-responsive	6	435 (320–570)		209	30 (14%)	
6–12 months	Sapropterin-responsive	8	370 (205–595)	<0.001	114	24 (21%)	0.123
	Sapropterin-non-responsive	6	540 (420–640)		135	40 (30%)	
12–24 months	Sapropterin-responsive	8	455 (223–638)	<0.001	146	40 (27%)	<0.001
	Sapropterin-non-responsive	6	600 (470–750)		325	154 (47%)	

^1^ Mann–Whitney U test. ^2^ Chi-square test. ^3^ Therapeutic upper target range: 360 µmol/L for <12 years; 600 µmol/L for ≥12 years [37].

**Table 2:** Prescribed protein intake and anthropometric outcomes in sapropterin-responsive and non-responsive children at baseline and 24 months.

Prescribed Protein	Sapropterin-Responsive (*n* = 20) *			Sapropterin-Non-Responsive (*n* = 12)			Between-Group *p* ^2^
	Baseline Median (Q1–Q3)	24 Months Median (Q1–Q3)	Within-Group *p* ^1^	Baseline Median (Q1–Q3)	24 Months Median (Q1–Q3)	Within-Group *p* ^1^	
Total protein (g/day)	67 (64–76)	75 (70–84)	0.103	68 (63–84)	84 (65–87)	0.213	0.74
Total protein (g/kg/day)	1.7 (1.5–2.4)	1.7 (1.4–2.4)	0.881	2.2 (1.2–2.7)	1.2 (1.1–2.0)	0.142	0.08
Natural protein (g/day)	10 (6–13)	28 (20–40)	<0.001	7 (4–14)	6 (4–8)	0.097	<0.001
Natural protein (g/kg/day)	0.2 (0.1–0.4)	0.8 (0.3–1.1)	<0.001	0.2 (0.1–0.4)	0.1 (0.1–0.1)	0.017	<0.001
Protein equivalent from protein substitutes (g/day)	60 (56–63)	45 (40–60)	0.026	60 (49–65)	80 (60–80)	0.123	<0.001
Protein equivalent from protein substitutes (g/kg/day)	1.5 (1.3–2.1)	1.2 (0.8–1.6)	0.061	1.6 (1.1–2.6)	1.1 (1.0–1.8)	0.328	0.53

^1^ Within-group comparisons between baseline and 24 months were analyzed using the Wilcoxon signed-rank test. ^2^ Between-group comparisons at 24 months were analyzed using the Mann–Whitney U test. * One sapropterin-responsive child who was non-compliant and discontinued the diet was excluded from the protein intake analysis.

**Table 3:** Anthropometric outcomes in sapropterin-responsive and non-responsive children at baseline and 24 months.

Anthropometry	Sapropterin-Responsive (*n* = 21)			Sapropterin-Non-Responsive (*n* = 12)			Between-Group *p* ^2^
	Baseline Mean ± SD	24 Months Mean ± SD	Within-Group *p* ^1^	Baseline Mean ± SD	24 Months Mean ± SD	Within-Group *p* ^1^	
Weight z-score	0.7 ± 1.3	0.8 ± 1.3	0.24	1.5 ± 1.0	1.7 ± 1.0	0.42	0.037
Height z-score	0.4 ± 0.9	0.6 ± 1.0	0.03	0.6 ± 0.9	0.3 ± 1.0	0.19	0.515
BMI z-score	0.6 ± 1.4	0.6 ± 1.3	0.94	1.5 ± 1.3	1.7 ± 1.2	0.09	0.026

^1^ Within-group comparisons between baseline and 24 months were analyzed using the paired *t*-test. ^2^ Between-group comparisons at 24 months were analyzed using the independent *t*-test.

**Table 4:** Frequency of consumption (portions per week) of selected regular and low-protein foods among sapropterin-responsive and non-responsive children at baseline and 24 months.

	Sapropterin-Responsive (*n* = 21)					Sapropterin-Non-Responsive (*n* = 12)				
	Baseline		24 Months		Within-Group *p*	Baseline		24 Months		Within-Group *p*
	Median (Q1–Q3)	Consuming n (%)	Median (Q1–Q3)	Consuming n (%)		Median (Q1–Q3)	Consuming n (%)	Median (Q1–Q3)	Consuming n (%)	
Food Item (portion/week)										
Cow’s milk (30 mL)	0 (0–0)	4 (19%)	0 (0–7)	9 (43%)	0.009	0 (0–0)	0 (0%)	0 (0–0)	0 (0%)	N/A
Low protein milk (200–250 mL)	7 (1–11)	18 (86%)	2 (0–7)	13 (62%)	0.001	7 (2–9)	11 (92%)	5 (4–8)	11 (92%)	1
Regular cheese (15–20 g)	0 (0–0)	3 (14%)	0 (0–3)	10 (48%)	0.009	0 (0–0)	0 (0%)	0 (0–0)	0 (0%)	N/A
Low protein cheese (20 g)	3 (2–5)	16 (76%)	0 (0–2)	9 (43%)	0.004	9 (4–14)	11 (92%)	6 (4–16)	11 (92%)	0.894
Meat/fish/eggs (60–80 g, 1 medium egg)	0 (0–0)	5 (24%)	2 (0–5)	11 (52%)	0.004	0 (0–0)	0 (0%)	0 (0–0)	0 (0%)	N/A
Low protein meat/fish/eggs (80 g)	1 (0–3)	12 (57%)	1 (0–3)	11 (52%)	0.797	4 (2–7)	10 (83%)	3 (1–8)	9 (75%)	0.623
Regular bread (30–70 g)	0 (0–0)	5 (24%)	8 (3–11)	17 (81%)	0.013	0 (0–0)	0 (0%)	0 (0–0)	0 (0%)	N/A
Low protein bread (30–70 g)	2 (0–7)	14 (67%)	0 (0–0)	5 (24%)	0.008	20 (9–27)	12 (100%)	16 (7–27)	10 (83%)	1
Regular pasta (15 g cooked)	0 (0–2)	8 (38%)	3 (2–10)	19 (90%)	0.001	0 (0–2)	5 (42%)	0 (0–1)	4 (33%)	0.203
Low protein pasta (80–100 g cooked)	2 (2–5)	18 (86%)	0 (0–1)	9 (43%)	0.003	4 (3–7)	12 (100%)	4 (3–6)	12 (100%)	0.413
Chips and processed potatoes (30–55 g)	5 (3–10)	20 (95%)	6 (3–15)	21 (100%)	0.153	5 (3–6)	11 (92%)	4 (4–9)	11 (92%)	1
Vegetables (20–60 g)	11 (5–24)	18 (86%)	14 (8–17)	19 (90%)	0.563	13 (9–19)	12 (100%)	13 (10–14)	12 (100%)	0.359
Fruit (fresh) (80–100 g)	7 (1–14)	16 (76%)	7 (2–7)	18 (86%)	0.2	14 (6–16)	11 (92%)	4 (0–7)	7 (58%)	0.05

Within-group changes were assessed using the Wilcoxon signed-rank test. N/A: not applicable (no consumption in either period).

**Table 5:** Impact on Family Scale scores in sapropterin-responsive and non-responsive children at baseline and 24 months.

Subscale	Sapropterin-Responsive (*n* = 21)			Sapropterin Non-Responsive (*n* = 12)			Between-Group *p* ^2^
	Baseline Mean ± SD	24 Months Mean ± SD	Within-Group *p* ^1^	Baseline Mean ± SD	24 Months Mean ± SD	Within-Group *p* ^1^	
Financial Impact	7.8 ± 3	10.3 ± 3.1	0.017	9.1 ± 3.2	8.3 ± 1.8	0.481	0.06
Familial-Social Impact	16.8 ± 5.9	24 ± 4.7	<0.001	19.1 ± 5.4	20 ± 2.7	0.699	0.01
Personal Strain	13.4 ± 5	18 ± 5.9	0.003	15.2 ± 3.4	17 ± 1.8	0.04	0.48
Mastery	8.5 ± 2.7	8.6 ± 3	0.602	10.3 ± 1.6	10.1 ± 3.4	0.882	0.21
Total Score	46.5 ± 12.8	60.9 ± 12.2	<0.001	53.6 ± 9.7	55.4 ± 3.7	0.614	0.07

^1^ Within-group comparisons between baseline and 24 months were analyzed using paired *t*-test. ^2^ Between-group comparisons at 24 months were analyzed using the independent *t*-test.

**Table 6:** Neophobia Questionnaire and HADS scores in sapropterin-responsive and non-responsive children at baseline and 24 months.

Measure	Sapropterin-Responsive (*n* = 21)			Sapropterin Non-Responsive (*n* = 12)			Between-Group *p* ^2^
	Baseline Mean ± SD	24 Months Mean ± SD	Within-Group *p* ^1^	Baseline Mean ± SD	24 Months Mean ± SD	Within-Group *p* ^1^	
Neophobia Questionnaire							
Food Neophobia	28.5 ± 10.3	36.3 ± 11.7	0.005	34.2 ± 14.5	37.6 ± 11.3	0.295	0.759
General Neophobia	14.5 ± 8.6	18.6 ± 9.1	0.091	16.9 ± 8.8	18.1 ± 8.5	0.531	0.88
	Median (Q1–Q3)	Median (Q1–Q3)	Within-group *p* ^1^	Median (Q1–Q3)	Median (Q1–Q3)	Within-group *p* ^1^	Between-group *p* ^2^
HADS							
Anxiety score (caregivers + adolescents)	8 (5–15)	8 (3–12)	0.087	6 (5–10)	6 (6–8)	1	0.77
Depression score (caregivers + adolescents)	4 (2–7)	2 (0–4)	0.099	3 (2–5)	4 (2–5)	0.877	0.33
Anxiety score (caregivers)	8 (5–14)	7 (3–12)	0.232	8 (6–10)	7 (5–8)	0.784	0.97
Depression score (caregivers)	4 (2–7)	1 (0–3)	0.013	4 (2–7)	2 (0–4)	0.104	0.64
Anxiety score (adolescents ≥ 12 years)	6 (5–15)	8 (5–10)	0.291	5 (4–6)	6 (6–8)	0.854	0.61
Depression score (adolescents ≥ 12 years)	4 (2–7)	4 (0–10)	0.932	2 (2–4)	5 (4–5)	0.057	0.79

^1^ Within-group comparisons between baseline and 24 months were analyzed using the Wilcoxon signed-rank. ^2^ Between-group comparisons at 24 months were analyzed using the Mann–Whitney U test. HADS: Hospital Anxiety and Depression Scale. Interpretation: normal = 0–7; borderline abnormal = 8–10; abnormal = 11–21.

**Table 7:** Themes reflecting the experiences of caregivers at 24-month follow-up.

Theme	Sapropterin Responsive (*n* = 21)	Sapropterin Non-Responsive (*n* = 12)
Emotional response to result	Positive, life-changing; increased energy and concentration: 95% (20/21)	Disappointed then accepting; hopeful for future options: 100% (12/12)
Dietary flexibility and spontaneity	Increased food variety and freedom: 100% (21/21)	No dietary change; restrictions persist: 100% (12/12)
Time burden (shopping/preparation/cooking)	Reduced to typical household level: 86% (18/21)	High and ongoing; detailed planning required: 100% (12/12)
Special low-protein foods	Reduced or minimal use: 67% (14/21)	Heavy daily dependence: 100% (12/12)
Protein substitutes	Quick (<1 min), minimal issues: 86% (18/21); 2 stopped (1 nonadherent, 1 no longer required)	Quick but more issues: 42% (5/12) (resistance when unwell)
Food expenditure	Lower or similar: 67% (14/21); higher due to appetite or takeaways: 24% (5/21)	High or unchanged: 75% (9/12)
Eating out (frequency and conflict)	Frequent (weekly/monthly): 100% (21/21); conflicts rare: 14% (3/21)	Infrequent: 25% (3/12); conflicts more common: 25% (3/12)
Holidays (preference and planning)	UK and abroad: 48% (10/21); rarely due to autism or financial limits: 24% (5/21); minimal adaptations	UK and abroad: 58% (7/12); planning and organization required
School food provision *	Packed lunches: 72% (13/18); school meals: 28% (5/18)	Packed lunches: 67% (8/12)
Protein substitute use at school ^†^	Consistent use: 76% (13/17)	Consistent use: 100% (12/12)
Social participation and sleepovers	Attended parties or food-related events with minimal restrictions: 86% (18/21); sleepovers allowed: 62% (13/21)	Attended occasionally, often conditional or requiring planning: 67% (8/12); sleepovers allowed: 33% (4/12)

* Calculated among school-attending participants. ^†^ Calculated among school-attending participants using protein substitutes.

## Data Availability

The original contributions presented in this study are included in the article. Further inquiries can be directed to the corresponding author.

## Supplementary Materials

The following supporting information can be downloaded at: https://www.mdpi.com/article/10.3390/nu18030446/s1, Supplementary Table S1: PKU variants, variant classification, and natural protein tolerance at 24 months.

## Abbreviations

The following abbreviations are used in this manuscript:

BH4	Tetrahydrobiopterin
BIMDG	The British Inherited Metabolic Disease Group
DHPR	Dihydropteridine Reductase
EMA	European Medicines Agency
FDA	U.S. Food and Drug Administration
FFQ	Food Frequency Questionnaire
HADS	Hospital Anxiety and Depression Scale
NHS	National Health Service
PAH	Phenylalanine Hydroxylase
Phe	Phenylalanine
PKU	Phenylketonuria
UK	United Kingdom
