# Supplementary material for: Two-Year Outcomes of Sapropterin Treatment in Children with Phenylketonuria: A Longitudinal Observational Study of Metabolic, Dietary, and Psychosocial Effects
Source: Nutrients. 2026 Jan 29;18(3):446. doi: 10.3390/nu18030446 (PMC12899584; doi:10.3390/nu18030446)
Supplement: Supplementary file 1 [file nutrients-18-00446-s001.zip › nutrients-4095443-supplementary.pdf]

Supplementary Table S1. PKU variants, variant classification, and natural protein tolerance at 24 months.

Patient	Affected gene	PKU variants	Classification based on Phe tolerance	Natural protein tolerance (g/day, 24 months)
		Sapropterin-responsive group (*n* = 21)		
1	PAH	c.194T>C p.(Ile65Thr); c.194T>C p.(Ile65Thr);	Mild	55
2	PAH	c.1241A>G p.(Tyr414Cys); c.1315+1G>A.?	Mild	30
3	PAH	c.194T>C p.(Ile65Thr) ; c. 1066-3C>T p?	Classical	22
4	PAH	c.926C>T p.(Ala309Val) ; c.1103A>G p.(Glu368Gly)	Classical	10
5	PAH	c. 1042C>G p. (Leu348Val) ; c.1066-3C>T p. ?	Mild	65
6	PAH	Not done	Classical	15
7	PAH	Not done	Classical	25
8	PAH	c. 1222 C>T p.(Arg 408 Trp) ; c706+519T>C	Mild	40
9	PAH	c.117C>G p.Phe.39Leu ; c.194T>C p.(Ile65Thr)	Classical	30
10	PAH	C1315+1G>A.p.? ; c.782G>A p(Arg261Gln)	Classical	14
11	PAH	c.194T>C p.(Ile65Thr); c.1042C>G p.(Leu348Val)	Mild	21
12	PAH	Not done	Classical	13
13	PAH	c.727C>T p.(Arg243*); c.782G>A p.(Arg261Gln) ;	Classical	17
14	PAH	c.1110A>G; p.(Glu370Gly) ; c.1110A>G; p.(Glu370Gly)	Mild	58
15	DHPR	Not applicable	DHPR Deficiency	30
16	PAH	Not done	Classical	40
17	PAH	c.1241A>G p.(Tyr414Cys); c.1315+1G>A.?	Mild	39
18	PAH	C194t>c ; c.1222C>T p.(Arg408Trp)	Classical	22
19	PAH	c.1042c>G; c1222c>T?	Classical	22
20	PAH	c.194T>C p.(Ile65Thr) ; C.1066-11G>Ap.?	Classical	Non adherence
21	DHPR	Not applicable	DHPR Deficiency	40
		Sapropterin non-responsive group (*n* = 12)		
22	PAH	c.1222C>T p.(Arg408Trp); c.1222C>T p.(Arg408Trp)	Classical	5
23	PAH	c.1315+1G>A p.? ; c.1315+1G>A p.?	Classical	4
24	PAH	c.1222 C>T p.Arg408Trp; c.1222 C>T p.Arg408Trp	Classical	7
25	PAH	c.1315+1G>A p.? ; c.1315+1G>A p.?	Classical	8
26	PAH	c.1222C>T p.(Arg408Trp); c.1222C>T p.(Arg408Trp)	Classical	4.5
27	PAH	c.558_559del p.(Trp187Glyfs*12); c.558_559del p.(Trp187Glyfs*12)	Classical	4
28	PAH	c.558_559del p.(Trp187Glyfs*12); c.558_559del p.(Trp187Glyfs*12)	Classical	4
29	PAH	c. 912+1 G>A p. ? ; c. 912+1 G>A p. ?	Classical	7.5
30	PAH	c.1315+1G>A.p.? ; c.1315+1G>A.p.?	Classical	7.5
31	PAH	c.1315+1G>A p.? ; c.1315+1G>A p.?	Classical	4
32	PAH	c.47_48del p.(Ser16*) ; c.47_48del p.(Ser16*)	Classical	8
33	PAH	c.1315+1G>A p.?; c.1315+1G>A p.?	Classical	9
